# Dental Research Data Availability and Quality According to the FAIR Principles

**DOI:** 10.1177/00220345221101321

**Published:** 2022-06-02

**Authors:** S.E. Uribe, A. Sofi-Mahmudi, E. Raittio, I. Maldupa, B. Vilne

**Affiliations:** 1 Bioinformatics Lab, Riga Stradins University, Riga, Latvia; 2 Department of Conservative Dentistry and Oral Health, Riga Stradins University, Riga, Latvia; 3 School of Dentistry, Universidad Austral de Chile, Valdivia, Chile; 4 Baltic Biomaterials Centre of Excellence, Riga Technical University, Riga, Latvia; 5 Seqiz Health Network, Kurdistan University of Medical Sciences, Seqiz, Kurdistan; 6 Cochrane Iran Associate Centre, National Institute for Medical Research Development, Tehran, Iran; 7 Institute of Dentistry, University of Eastern Finland, Kuopio, Finland

**Keywords:** deep learning, machine learning, open data, dental informatics, electronic dental records, outcomes research

## Abstract

According to the FAIR principles, data produced by scientific research should be findable, accessible, interoperable, and reusable—for instance, to be used in machine learning algorithms. However, to date, there is no estimate of the quantity or quality of dental research data evaluated via the FAIR principles. We aimed to determine the availability of open data in dental research and to assess compliance with the FAIR principles (or FAIRness) of shared dental research data. We downloaded all available articles published in PubMed-indexed dental journals from 2016 to 2021 as open access from Europe PubMed Central. In addition, we took a random sample of 500 dental articles that were not open access through Europe PubMed Central. We assessed data sharing in the articles and compliance of shared data to the FAIR principles programmatically. Results showed that of 7,509 investigated articles, 112 (1.5%) shared data. The average (SD) level of compliance with the FAIR metrics was 32.6% (31.9%). The average for each metric was as follows: findability, 3.4 (2.7) of 7; accessibility, 1.0 (1.0) of 3; interoperability, 1.1 (1.2) of 4; and reusability, 2.4 (2.6) of 10. No considerable changes in data sharing or quality of shared data occurred over the years. Our findings indicated that dental researchers rarely shared data, and when they did share, the FAIR quality was suboptimal. Machine learning algorithms could understand 1% of available dental research data. These undermine the reproducibility of dental research and hinder gaining the knowledge that can be gleaned from machine learning algorithms and applications.

## Introduction

Data-driven dentistry integrates the multitude of data sources available at the levels of the individual (e.g., clinical records and wearable devices), setting (e.g., geospatial, provider-related data), and system (e.g., insurance, regulatory and legislative data) that affect clinical care processes (Schwendicke and Krois 2022). Increased data availability combined with the ability to process them and the application of systems biology are transforming health care into proactive P4 medicine—that is, predictive, preventive, personalized, and participatory (Hood and Flores 2012). Scientific publications contain the processed data summary. By dental research data, we refer to raw data collected by the study but not transformed or analyzed (Lavrakas 2008). Although in the United States, the National Institute of Dental and Craniofacial Research (2022) provides $337.2 million annually for dental research grants supporting 728 projects, open dental data sets are still rare. In the cases where dental data are available, the data are complex (Tachalov et al. 2021), have restricted access (Walji et al. 2022), or are in formats that limit their secondary use (Liu et al. 2013; Morris 2019). Currently, machine learning (ML) algorithms (a subset of artificial intelligence) can generate further output data that may differ from the initial input data. For example, ML can create knowledge from big data—for example, detecting subtle changes in patterns that can predict pathologic changes before humans can detect them (Ardila et al. 2019)—or find complex relationships between large amounts of data and multidimensional variables (Uddin et al. 2019). Thus, ML-based techniques might provide a deeper and more detailed understanding of the complex interplay of factors that determine the oral health of individuals and communities, fostering new diagnostic, treatment, and prognostic techniques.

To enable ML-based techniques, research data need to be distributed so that machines can understand them. However, the raw data generated by research are rarely shared (Miyakawa 2020). A large part of these valuable and quality clinical data is lost or kept without being reused (Baker 2016). In 2016, a diverse set of stakeholders from academia, industry, funding agencies, and publishers agreed on a set of principles to integrate big data analytics and artificial intelligence tools for scientific development (Wilkinson et al. 2016). Thus, the FAIR principles were established, specifying that data produced by scientific research be findable, accessible, interoperable, and reusable (Wilkinson et al. 2016). The FAIR principles represent “domain-independent, high-level principles that can be applied to a wide range of scholarly outputs” (e.g., research data; Wilkinson et al. 2016). The FAIR data principles were designed for machines to understand and process data (i.e., machine-actionable operations generated by research). Most research data are now produced by human researchers, and they must translate their results into a language that machines can understand. This process requires converting complex concepts into snippets of information that allow machines to connect them into networks and find patterns. Currently, a consensus has been reached on how the “FAIRness” of research data should be evaluated (Bahim et al. 2020), and validated tools have been developed to assess the FAIR metrics of a given research data set objectively (Wilkinson et al. 2019). Yet, to date, there is no estimate of the quantity and quality of dental research data evaluated with the FAIR criteria to assess the availability for ML processing of dental research data sets. Therefore, the objective of this research is first to determine the availability of open data in dental research and then to evaluate the FAIRness of the shared dental research data to estimate the proportion of open research dental data that ML algorithms could process.

## Methods

This descriptive study was prepared with the STROBE guideline (von Elm et al. 2007). The protocol is available at OSF Registries (https://osf.io/zs5dk). We used the Royal Society’s (2012) definitions of an open access (OA) publication: the available publication of research papers so that anyone can access and reuse them and open data research (i.e., accessible, usable, and assessable data).

### Data Sources and Study Selection

For OA articles, we used the Europe PubMed Central (EPMC) database. As of January 2022, the EPMC contained 39.9 million abstracts and 7.5 million full-text articles from PubMed and PubMed Central (EPMC 2021). Thus, the EPMC database is a valuable source for data-driven bibliographic dental research. The dental journals were selected by a list of PubMed-indexed dental journals provided by the National Library of Medicine (2021; available in the Appendix). We included all articles published from 2016 to 2021 using the *europepmc* package in R (Levchenko et al. 2018). Only original scientific articles in English were included, excluding nonscientific articles, letters, and editorials. We downloaded all identified OA journal articles in full text from the EPMC and processed them with the *metareadr* R package (Serghiou et al. 2021). While we assumed that OA articles available in the EPMC were more likely to contain open data (Page et al. 2022), we also checked non-OA journals. Thus, we randomly selected 500 non-OA articles. We chose a sample size of 500 because it would provide an accurate estimate of data sharing in dental non-OA articles (2% to 3% margin of error and 95% confidence level), considering the prevalence of data sharing (<10%) in OA articles and the total number of non-OA articles identified in the preliminary search.

### Data Extraction

Data sharing in the retrieved OA dental articles (in XML format) and the 500 non-OA dental articles (in PDF format) was assessed programmatically with the *rtransparent* package (Serghiou et al. 2021). The text-mining search for open data sharing was done with the *oddpub* package in R (Riedel et al. 2020). Both packages identify whether a data/code-sharing statement is present, determine how data were shared, and extract the phrase in which this was detected. These text-mining algorithms detect data sharing where data were made available and circumvent claims of data sharing “upon request” or the equivalent.

### Variables

We extracted the DOI (digital object identifier) when the open data set was available in an external repository or when it used the same DOI of the publication where the data were available as supplementary material.

To assess the FAIRness of the shared data objects programmatically, we employed the FAIR specification version 0.3d (Wilkinson et al. 2019). The output of this tool provides 4 individual levels for each component of the FAIR principle (maximum points): findability (15), accessibility (9), interoperability (9), and reusability (15) for a maximum of 48 points (see Appendix).

### Bias

To reduce the risk of bias, we manually checked the programmatically selected items, finding that the accuracy of the algorithm was 98.8% (95% CI, 98.5% to 99.0%), with a sensitivity of 55.6% (95% CI, 48.4% to 62.7%) and a specificity 100% (95% CI, 99.9% to 100%). The compliance with the FAIR principles for the data sets was evaluated programmatically with independently validated algorithms (Koers et al. 2020).

### Analysis

We performed a descriptive analysis of compliance with FAIR maturity metrics for free data articles. FAIR-level differences between different journals and trend over time were explored visually. The R-script analysis is available at doi:10.5281/zenodo.6460190.

## Results

All extracted data were harmonized into a unified data set (see Zenodo data repository at doi:10.5281/zenodo.6460190).

The search of the EPMC database retrieved 7,049 dental OA articles from 76 dental journals. Of 500 random non-OA publications, we obtained the full text of 460 articles from 99 journals. From those 7,509 dental articles (OA + non-OA) with full texts, the text-mining algorithms indicated that 200 items were indexed by stating that they had data.

After manual removal of false positives (i.e., those that did not share data despite the algorithms indicating as much), the final number of articles with open data was 112 (1.5%), of which 109 were OA articles and 3 were non-OA. These false positives occurred because the articles mentioned that they had the data sets but did not identify any repository. Seven journals accounted for 84.2% of the open data publications: *BMC Oral Health* (60%), *Progress in Orthodontics* (6.2%), *International Journal of Implant Dentistry* (4.5%), *International Journal of Oral Science* (4.5%), *Clinical and Experimental Dental Research* (3.6%), *Journal of Dental Research* (2.7%), and *Journal of Clinical Periodontology* (2.7%). The remaining 144 journals had no open data available. The percentage of publications with open data available was 11.6% for 2016 to 2018 and 21.3% for 2019 to 2021. Table 1 shows details of the articles analyzed according to open data availability.

**Table 1.** Availability of Open Data by Journal and Year.

	Open Data Available, *n* (%)	
	No (*n* = 7,437)	Yes (*n* = 112)
Journal		
*BMC Oral Health*	1,724 (23)	67 (60)
*Prog Orthod*	253 (3.4)	7 (6.2)
*Int J Implant Dent*	350 (4.7)	5 (4.5)
*Int J Oral Sci*	198 (2.7)	5 (4.5)
*Clin Exp Dent Res*	387 (5.2)	4 (3.6)
*J Dent Res*	77 (1.0)	3 (2.7)
*J Clin Periodontol*	68 (0.9)	3 (2.7)
*Head Face Med*	194 (2.6)	2 (1.8)
*J Appl Oral Sci*	483 (6.5)	2 (1.8)
*Med Oral Patol Oral Cir Bucal*	639 (8.6)	2 (1.8)
*Mol Oral Microbiol*	12 (0.2)	2 (1.8)
*Cranio*	3 (<0.1)	1 (0.9)
*Eur J Oral Sci*	28 (0.4)	1 (0.9)
*Eur J Orthod*	22 (0.3)	1 (0.9)
*J Oral Pathol Med*	15 (0.2)	1 (0.9)
*J Oral Rehabil*	62 (0.8)	1 (0.9)
*J Orofac Orthop*	34 (0.5)	1 (0.9)
*J Periodontal Res*	30 (0.4)	1 (0.9)
*Odontology*	45 (0.6)	1 (0.9)
*Oral Dis*	86 (1.2)	1 (0.9)
*Periodontol 2000*	20 (0.3)	1 (0.9)
Other (144 journals)	2,707 (36.4)	0 (0)
Publication year		
2016	733 (9.9)	14 (12)
2017	815 (11)	8 (7.1)
2018	1,117 (15)	18 (16)
2019	1,018 (14)	19 (17)
2020	1,556 (21)	15 (13)
2021	2,195 (30)	38 (34)
2022	3 (<0.1)	0 (0)

Ordered by number of open data articles available.

The average (SD) level of compliance with FAIR metrics was 32.6% (31.9%). The average for each metric was as follows: findability, 3.4 (2.7) of 7; accessibility, 1.0 (1.0) of 3; interoperability, 1.1 (1.2) of 4; and reusability, 2.4 (2.6) of 10. The compliance by metric is shown in Table 2, which also details the results by journal (for those with >3 articles with open data) and by year. We detected no differences by journal or year.

**Table 2.** Summary of FAIR Metrics.

	*n*	Mean, %	SD, %
FAIRness	112	32.6	31.9
FAIR metrics			
Findability	112	48.6	38.6
Accessibility	112	31.7	33.3
Interoperability	112	27.0	30.0
Reusability	112	24.0	26.0
Journal			
*Prog Orthod*	7	41.4	35.5
*BMC Oral Health*	67	37.7	32.5
*Int J Implant Dent*	5	32.4	38.9
Other^a^	18	28.3	29.9
*Int J Oral Sci*	5	18.2	31.8
*Clin Exp Dent Res*	4	11.0	8.1
*J Clin Periodontol*	3	4.0	0.0
*J Dent Res*	3	4.0	0.0
Year			
2016	14	44.0	34.0
2017	8	31.8	34.8
2018	18	28.4	32.1
2019	19	35.1	33.6
2020	15	24.1	27.8
2021	38	32.5	32.0

FAIR, findable, accessible, interoperable, and reusable.

a Those with <3 articles.

**Figure 1.**
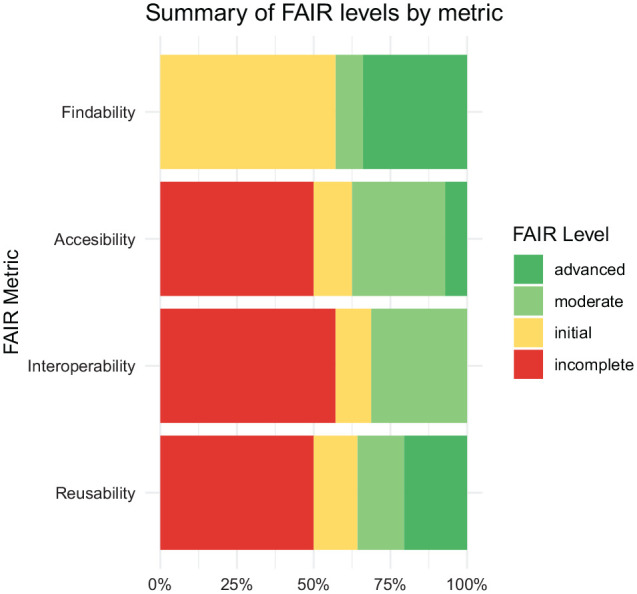
FAIR level by metric.

The percentages of moderate or advanced compliance were as follows: findability, 42.6%; accessibility, 37.5%; interoperability, 31.3%; and reusability, 35.7% (Fig. 1).

**Figure 2.**
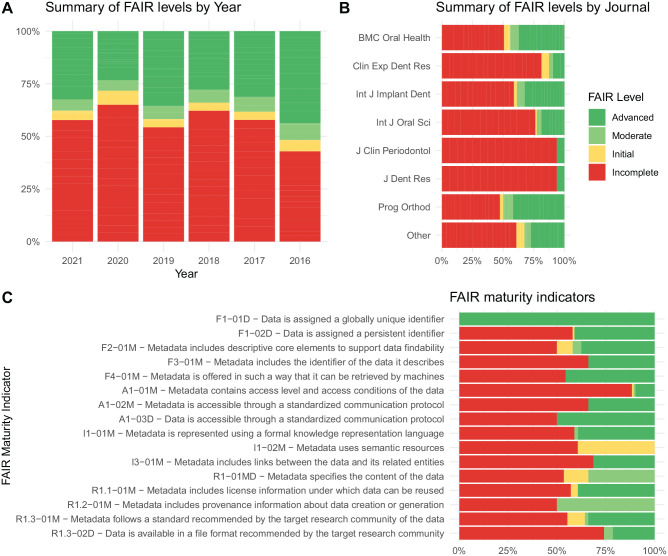
FAIR levels by (**A**) year and (**B**) journal. (**C**) FAIR maturity indicators. FAIR, findable, accessible, interoperable, and reusable.

The detail of compliance for each metric of maturity by journal and year is shown in Figure 2A and B, where the breakdown is by journal (those with <3 publications are grouped into “other”).

When the level of compliance with the FAIR maturity indices was examined, the one with the highest compliance at the advanced and moderate levels was “Data are assigned a globally unique identifier” (100%), followed by “Data are accessible through a standardized communication protocol” and “Metadata include provenance information about data creation or generation,” both with 50%. Maturity rates that had less compliance were “Metadata contain access level and access conditions of the data” (10.7%), “Data are available in a file format recommended by the target research community” (25.9%) and “Metadata include links between the data and their related entities” (31.2%; Fig. 2C).

## Discussion

We found that 1.5% of the publications had open data available and the proportion remained constant from 2016 to 2020 with an increase in 2021. Data sharing was more common in OA than in non-OA articles. Findability, accessibility, interoperability, and reusability of the shared data were often suboptimal, and no improvement occurred over the years. When compliance with the FAIR principles was evaluated, it was 32.6%, with low levels for all principles. Using the same programming algorithm, Serghiou et al. (2021) found that 68 (20%) of 349 biomedical articles available in PubMed had a data-sharing statement. The algorithm used to extract data availability obtained similar accuracy to that reported, with a propensity for false positives. Similar to Serghiou et al., Wallach et al. (2018) noted that 19 (18.3%) of 104 biomedical articles available in PubMed published between 2015 and 2017 had data available. However, while they cited an upward trend in the availability of research data, we found that the number of publications reporting open data in dental journals has remained constant at 1.5%. There are no previous publications about data quality based on the FAIR principles, given that the development of the programming algorithms is very recent. Hence, our results provide a first approximation of the quality of open dental research data available to date.

Overall, the finding that few research data are available in dentistry and that what is available is of low quality may have 2 significant consequences: the impact on reproducibility and the impact on ML applications. First, it means that the replicability of the available dental research results is limited and low. Low replicability implies that some results may contain errors or biases very difficult to detect without the original data. A survey of the statistical errors in microleakage studies in operative dentistry found that when the raw data are available for independent validation, the conclusions had to be altered for 15.4% of these reanalyzed studies (Lucena et al. 2011). However, despite their potential for improving and correcting scientific knowledge, these kinds of reanalyses are rarely conducted or published even if data are provided. Also, replicability increases confidence in the scientific process (National Academies of Sciences, Engineering, and Medicine 2019), which could serve to decrease propagation or limit the effects of misinformation. Second, our results provide a machine perspective on data availability. So far, data sharing has had little effect, as reanalysis by other researchers is rare (Vazquez et al. 2021). However, the advent of ML algorithms may change this situation, allowing the reuse of available data. While data management and dissemination are crucial to research, the development of ML algorithms has extended this stewardship with the concept of machine-actionability. FAIR requires that “the machine understands what we mean” in simple terms. Within the FAIR metrics, there are 2 critical indicators—“Metadata are offered in such a way that they can be retrieved by machines” and “Data are available in a file format recommended by the target research community”—that have the level of advanced/moderate at 45.5% and 33.9% of the available data. In other words, machines could understand and access <1% of the data generated by dental research. If the data were shared in a machine-understandable manner, it could be used, for example, to independently validate the performance of ML algorithms and detect potential biases by providing patient data in different locations for different procedures.

Our study has some limitations. Although we restricted the sample to journal articles classified by the EPMC, it may have included some articles (e.g., commentaries) that cannot be expected to share data. In addition, the performance of text-mining algorithms has not been explicitly validated with articles published in dental journals, and our small validation sample indicated that the algorithms may have under- or overestimated data sharing. Some articles stated that they had open data available but instead provided the PDFs of the peer review process, while others claimed to have data available but did not provide any other information on where to locate these data. These situations can be solved by adhering to a definition of research data or by the journals stating their requirements for peer review. A recent editorial by Schwendicke et al. (2022) offers some solutions: enforcing open data and code, realigning incentives for peer review, establishing standards and norms for data and data analysis, and encouraging authors to make data and code testable, even if not accessible. The fact that we found few publications with available data suggests that incentives for researchers should be realigned. For example, the Royal Society (2012) proposes, among other things, that “assessment of university research should reward the development of open data on the same scale as journal articles and other publications.” Finally, our research was limited to data sets associated with published research. However, there is potential for research on data available in open data sets, such as datasetsearch.research.google.com or Kaggle, or closed data sets, such as BigMouth Dental Data Repository (https://bigmouth.uth.edu/).

During the manual data examination, we found some that shared peer reviews as research data, while in other cases, we found repositories that contained patients’ personal information. In most cases, it was not possible to identify the data coding. Thus, good data management practices should be promoted in the training of researchers and their appropriate communication. What can a researcher do to improve the FAIRness of the data? While there are numerous FAIR research guidelines available, the highest-scoring article offers some practical and straightforward tips. Choi et al. (2020) shared the data associated with their publication through a repository (Kim et al. 2020), consisting of the spreadsheet of the raw data hosted in the general Zenodo repository. This repository automatically adds metadata that substantially improve the FAIRness of the data. In our experience with other repositories, not all automatically add these metadata, so choosing a repository is crucial. This data set could have increased its score if it had shared the data in an open format, such as comma-separated values, instead of a proprietary one, such as xlsx. However, having shared the raw data is already a significant advance. If there are apprehensions about disclosing personal information, software packages can perform this task, such as anonymizer for R (Hendricks 2015). In this regard, the Royal Society (2012) suggests that this issue can be improved on several levels. Universities should make data sharing the default policy, limiting the option not to share when it is not optimal for the return on public investment. Also, universities should consider the incentives and rewards for data sharing and publications at the same level.

At the researcher level, recent results show that researchers are willing to share data. A survey conducted by Spallek et al. (2019), all 42 dental researchers responded that data sharing should be promoted and facilitated. Also, 27 (64%) indicated that they have been required to share data through a data repository.

The major concerns are the protection of the participants’ data and doubts about the appropriate use of the data. Funding bodies in the United States (National Institutes of Health 2022) and Europe (European Research Council 2021) are starting to require publicly funded research to declare the data management plan and release the data. Thus, a recent initiative is the National Institutes of Health’s new Scientific Data Sharing website at https://sharing.nih.gov/. At the journal level, the move is toward adopting 1 of 3 strategies for data transparency rigor:

*Disclosure:* the article must state whether the data supporting the results are available.
*Mandate:* the article must deposit the data supporting the results in a trusted repository.
*Verify:* shared data must be made available to a third party to verify that data can be used to replicate findings in the article.

Additionally, Schwendicke et al. (2022) suggest that the code of the analyses be made available, a suggestion that we share. Schwendicke et al. suggest 5 possibilities: 1) enforce open data and open code; 2) realign incentives for peer review; 3) establish standards and norms for data and advanced data analyses in dentistry on which to build; 4) push for authors to make data and code testable, even if not accessible; and 5) engage additional reviewers. For data sets that can be used for ML but cannot be shared for confidentiality reasons, federated learning can also be used. Federated learning has emerged as a prospective solution that facilitates distributed collaborative learning without disclosing original training data (Truong et al. 2021).

Some disciplines are more open to sharing data—for example, climate science, where it is customary to contribute to large open data repositories (Grinspan and Worker 2021). Although there are privacy constraints in medicine, medical research data should be as open as possible and as closed as necessary (Landi et al. 2020). Accordingly, there are guidelines available that detail, among other benefits, improving the monitoring of drug safety and efficacy, accelerating innovation, and facilitating secondary data analysis to explore new scientific questions (Mello et al. 2013). Also, open science practices, such as data sharing following the FAIR principles, increase citizens’ trust in science, promoting their participation in scientific studies, data collection, and science funding (OECD 2018).

The availability of quality research data would increase confidence in results and encourage “informed users” to decrease the asymmetry of information among researchers, clinicians, and the general public. The use of the FAIR principles would also allow humans and machines to access research data, which would increase the tools available to explore the complex web of large multidimensional variables that explain people’s health. This machine-actionable research data would strengthen the development of data-driven dentistry and contribute to the ultimate goal of dentistry: to improve people’s health and quality of life.

## Supplemental Material

sj-rtf-1-jdr-10.1177_00220345221101321 – Supplemental material for Dental Research Data Availability and Quality According to the FAIR Principles Click here for additional data file. Supplemental material, sj-rtf-1-jdr-10.1177_00220345221101321 for Dental Research Data Availability and Quality According to the FAIR Principles by S.E. Uribe, A. Sofi-Mahmudi, E. Raittio, I. Maldupa and B. Vilne in Journal of Dental Research

## Author Contributions

S.E. Uribe, contributed to conception, design, data acquisition, analysis, and interpretation, drafted and critically revised the manuscript; A. Sofi-Mahmudi, E. Raittio, contributed to design, data acquisition, analysis, and interpretation, drafted and critically revised the manuscript; I. Maldupa, B. Vilne, contributed to conception and data interpretation, critically revised the manuscript. All authors gave final approval and agree to be accountable for all aspects of the work.
