# Supplementary material for: Dental Research Data Availability and Quality According to the FAIR Principles
Source: J Dent Res. 2022 Jun 2;101(11):1307–13. doi: 10.1177/00220345221101321 (PMC9516597; doi:10.1177/00220345221101321)
Supplement: sj-rtf-1-jdr-10.1177_00220345221101321 – Supplemental material for Dental Research Data Availability and Quality According to the FAIR Principles [file sj-rtf-1-jdr-10.1177_00220345221101321.rtf]

 
 
Supplementary material

Index
TABLE 1 SUPP. FAIR Metrics	1
DATASET AND ANALYSIS SCRIPT	5
DENTAL JOURNALS LIST	6


TABLE 1 SUPP. FAIR Metrics
Supplementary material from Quality of Dental Research Data According to FAIR (Findability, Accessibility, Interoperability, Reusability) Principles by Uribe, Sofi-Mahmudi, Raittio, Maldupa, and Vilne
Source: https://github.com/FAIRMetrics/Metrics/blob/master/Metrics-1.0.3.zip 
Authors: Mark D. Wilkinson, Susanna-Assunta Sansone, Erik Schultes, Peter Doorn, Luiz Olavo Bonino da Silva Santos, Michel Dumontier . 
FAIRMetrics/Metrics is licensed under the MIT License.  

Principle	Metrics	Practical Tests	
F1	FsF-F1-01D
Data is assigned a globally unique identifier	•	The object is identified by an unique identifier (GUID or IRI) that follows a proper syntax.
•	The identifier is web-accessible (not broken).	
F1	FsF-F1-02D
Data is assigned a persistent identifier	•	A data identifier is specified based on a commonly accepted persistent identifier scheme suitable for research data.
•	The identifier is web-accessible, i.e., it resolves to a landing page with metadata of the data object.	
F2	FsF-F2-01M
Metadata includes descriptive core elements to support data findability.	•	Some metadata (at all) has been made available via common (web) standards.
•	Minimum core citation metadata is specified ( creator, title, publication date, publisher, and identifier)
•	Minimum core descriptive metadata is specified (creator, title, publisher, publication date, summary, keywords, identifier) through appropriate metadata fields.	
F3	FsF-F3-01M
Metadata includes the identifier of the data it describes.	•	Metadata contains a PID or URL which indicates the location of the downloadable data content
•	A data identifier is included in the metadata and it matches the identifier provided as part of the assessment request.	
F4	FsF-F4-01M
Metadata is offered in such a way that it can be retrieved by machines.	•	Metadata of the object is retrievable programmatically through at least one of the following methods:
•	Structured data embedded in the landing page of the data object.
•	Typed Links of metadata document or Signposting header links.
•	Content negotiation with a PID provider service (e.g., DataCite).	
A1	FsF-A1-01M
Metadata contains access level and access conditions of the data.	•	Metadata includes the level of data access (e.g., public, embargoed, restricted) and its access conditions using appropriate metadata fields.
•	Access level metadata is machine-readable, and this is verified against controlled vocabularies:
•	http://vocabularies.coar-repositories.org/documentation/access_rights/
•	http://purl.org/eprint/accessRights
•	http://publications.europa.eu/resource/authority/access-right	
A1	FsF-A1-02M
Metadata is accessible through a standardized communication protocol	•	The metadata URI’s scheme is based on a common application protocol.
•	The metadata is accessible through the identifier provided.	
A1	FsF-A1-03D
Data is accessible through a standardized communication protocol	•	The data URI’s scheme is based on a shared application protocol.
•	The data is accessible through the identifier provided.	
A2	FsF-A2-01M
Metadata remains available, even if the data is no longer available.	•	Programmatic assessment of the preservation of metadata of a data object can only be tested if the object is deleted or replaced. So this test is only applicable for deleted, replaced or obsolete objects. Importantly, continued access to metadata depends on a data repository’s preservation practice. Therefore, we regard that the assessment of metric applies to at the level of a repository, not at the level of individual objects. For this reason, we excluded its assessment details from the F-UJI implementation.	
I1	FsF-I1-01M
Metadata is represented using a formal knowledge representation language.	•	The metadata of the object is available in a formal knowledge representation language, e.g., through at least one of the following mechanisms:
•	Parsable, structured data is embedded in the landing page
•	Parsable, formal metadata (e.g., RDF, JSON-LD) is accessible through content negotiation, typed links or sparql endpoint.	
I1	FsF-I1-02M
Metadata uses semantic resources.	•	Namespaces of known semantic resources are present in the metadata of an object; exclude common namespaces (e.g., rdf, rdfs, xsd, owl) from the test.	
I3	FsF-I3-01M
Metadata includes links between the data and its related entities.	•	Metadata captures the relation between a data object and its related entity.
•	The relation is expressed using a relation type according to PROV-O or DataCite relation types.
•	Relations are preferably be expressed as actionable unique or persistent identifiers e.	
R1	FsF-R1-01MD
Metadata specifies the content of the data.	•	Metadata includes the type of the object and the technical properties of its data file such as format, size, observed or measured variables.
•	Metadata values of the properties comply with the actual data file.	
R1.1	FsF-R1.1-01M
Metadata includes license information under which data can be reused.	•	Metadata contains license information represented using an appropriate metadata element.
•	A standard, machine readable license is specified.	
R1.2	FsF-R1.2-01M
Metadata includes provenance information about data creation or generation.	•	Metadata includes properties representing data creation such as creator, contributors, creation and modification dates and version, source, and relations that indicate data creation activities.
•	Provenance metadata is available in a machine-readable version of PROV-O or PAV.	
R1.3	FsF-R1.3-01M
Metadata follows a standard recommended by the target research community of the data.	•	Metadata is available through at least one of the domain metadata standards listed in RDA Metadata Standards Catalog[6].	
R1.3	FsF-R1.3-02D
Data is available in a file format recommended by the target research community.	•	Data is available in a long-term file format as defined in ISO/TR 22299 
•	Data is available in an open format (see e.g., https://en.wikipedia.org/wiki/List_of_open_formats)
•	Data is available in a scientific file format (see e.g., Library of Congress dataset formats, Wolfram Alpha supported file formats)	


DATASET AND ANALYSIS SCRIPT
Sergio E Uribe, Ahmad Sofi-Mahmudi, Eero Raittio, Ilze Maldupa, & Baiba Vilne. (2022). Dataset Dental research data availability and quality according to FAIR principles (1.0) [Data set]. Zenodo. https://doi.org/10.5281/zenodo.6460190

URL: https://zenodo.org/record/6460190 
DENTAL JOURNALS LIST
“Acta Odontol Latinoam”[Journal] OR
“Acta Odontol Scand”[Journal] OR
“Adv Dent Res”[Journal] OR
“Am J Dent”[Journal] OR
“Am J Orthod Dentofacial Orthop”[Journal] OR
“Anesth Prog”[Journal] OR
“Angle Orthod”[Journal] OR
“Arch Oral Biol”[Journal] OR
“Atlas Oral Maxillofac Surg Clin North Am”[Journal] OR
“Aust Dent J”[Journal] OR
“Aust Endod J”[Journal] OR
“BMC Oral Health”[Journal] OR
“Br Dent J”[Journal] OR
“Br J Oral Maxillofac Surg”[Journal] OR
“Braz Dent J”[Journal] OR
“Braz Oral Res”[Journal] OR
“Bull Tokyo Dent Coll”[Journal] OR
“Can J Dent Hyg”[Journal] OR
“Caries Res”[Journal] OR
“Chin J Dent Res”[Journal] OR
“Cleft Palate Craniofac J”[Journal] OR
“Clin Adv Periodontics”[Journal] OR
“Clin Exp Dent Res”[Journal] OR
“Clin Implant Dent Relat Res”[Journal] OR
“Clin Oral Implants Res”[Journal] OR
“Clin Oral Investig”[Journal] OR
“Community Dent Health”[Journal] OR
“Community Dent Oral Epidemiol”[Journal] OR
“Compend Contin Educ Dent”[Journal] OR
“Cranio”[Journal] OR
“Dent Clin North Am”[Journal] OR
“Dent Mater J”[Journal] OR
“Dent Mater”[Journal] OR
“Dent Med Probl”[Journal] OR
“Dent Traumatol”[Journal] OR
“Dental Press J Orthod”[Journal] OR
“Dentomaxillofac Radiol”[Journal] OR
“Eur Arch Paediatr Dent”[Journal] OR
“Eur Endod J”[Journal] OR
“Eur J Dent Educ”[Journal] OR
“Eur J Oral Sci”[Journal] OR
“Eur J Orthod”[Journal] OR
“Eur J Paediatr Dent”[Journal] OR
“Eur J Prosthodont Restor Dent”[Journal] OR
“Evid Based Dent”[Journal] OR
“Facial Plast Surg”[Journal] OR
“Gen Dent”[Journal] OR
“Gerodontology”[Journal] OR
“Head Face Med”[Journal] OR
“Hua Xi Kou Qiang Yi Xue Za Zhi”[Journal] OR
“Indian J Dent Res”[Journal] OR
“Int Dent J”[Journal] OR
“Int Endod J”[Journal] OR
“Int J Comput Dent”[Journal] OR
“Int J Dent Hyg”[Journal] OR
“Int J Esthet Dent”[Journal] OR
“Int J Implant Dent”[Journal] OR
“Int J Oral Implantol (Berl)”[Journal] OR
“Int J Oral Maxillofac Implants”[Journal] OR
“Int J Oral Maxillofac Surg”[Journal] OR
“Int J Oral Sci”[Journal] OR
“Int J Paediatr Dent”[Journal] OR
“Int J Periodontics Restorative Dent”[Journal] OR
“Int J Prosthodont”[Journal] OR
“Int Orthod”[Journal] OR
“J Adhes Dent”[Journal] OR
“J Am Dent Assoc”[Journal] OR
“J Appl Oral Sci”[Journal] OR
“J Can Dent Assoc”[Journal] OR
“J Clin Dent”[Journal] OR
“J Clin Orthod”[Journal] OR
“J Clin Pediatr Dent”[Journal] OR
“J Clin Periodontol”[Journal] OR
“J Contemp Dent Pract”[Journal] OR
“J Craniofac Surg”[Journal] OR
“J Craniomaxillofac Surg”[Journal] OR
“J Dent Child (Chic)”[Journal] OR
“J Dent Educ”[Journal] OR
“J Dent Hyg”[Journal] OR
“J Dent Res”[Journal] OR
“J Dent”[Journal] OR
“J Endod”[Journal] OR
“J Esthet Restor Dent”[Journal] OR
“J Evid Based Dent Pract”[Journal] OR
“J Forensic Odontostomatol”[Journal] OR
“J Hist Dent”[Journal] OR
“J Indian Prosthodont Soc”[Journal] OR
“J Indian Soc Pedod Prev Dent”[Journal] OR
“J Int Acad Periodontol”[Journal] OR
“J Oral Biosci”[Journal] OR
“J Oral Facial Pain Headache”[Journal] OR
“J Oral Implantol”[Journal] OR
“J Oral Maxillofac Surg”[Journal] OR
“J Oral Pathol Med”[Journal] OR
“J Oral Rehabil”[Journal] OR
“J Oral Sci”[Journal] OR
“J Orofac Orthop”[Journal] OR
“J Orthod”[Journal] OR
“J Periodontal Res”[Journal] OR
“J Periodontol”[Journal] OR
“J Prosthet Dent”[Journal] OR
“J Prosthodont Res”[Journal] OR
“J Prosthodont”[Journal] OR
“J Public Health Dent”[Journal] OR
“J Stomatol Oral Maxillofac Surg”[Journal] OR
“J Vet Dent”[Journal] OR
“J World Fed Orthod”[Journal] OR
“JDR Clin Trans Res”[Journal] OR
“Med Oral Patol Oral Cir Bucal”[Journal] OR
“Minerva Dent Oral Sci”[Journal] OR
“Mol Oral Microbiol”[Journal] OR
“Monogr Oral Sci”[Journal] OR
“Ned Tijdschr Tandheelkd”[Journal] OR
“Odontology”[Journal] OR
“Oper Dent”[Journal] OR
“Oral Dis”[Journal] OR
“Oral Health Prev Dent”[Journal] OR
“Oral Maxillofac Surg Clin North Am”[Journal] OR
“Oral Maxillofac Surg”[Journal] OR
“Oral Surg Oral Med Oral Pathol Oral Radiol”[Journal] OR
“Orthod Craniofac Res”[Journal] OR
“Orthod Fr”[Journal] OR
“Pediatr Dent”[Journal] OR
“Periodontol 2000”[Journal] OR
“Prim Dent J”[Journal] OR
“Prog Orthod”[Journal] OR
“Quintessence Int”[Journal] OR
“Shanghai Kou Qiang Yi Xue”[Journal] OR
“Spec Care Dentist”[Journal] OR
“Stomatologiia (Mosk)”[Journal] OR
“Stomatologija”[Journal] OR
“Swiss Dent J”[Journal] OR
“Zhonghua Kou Qiang Yi Xue Za Zhi”[Journal]
